# The metastatic infiltration at the metastasis/brain parenchyma-interface is very heterogeneous and has a significant impact on survival in a prospective study

**DOI:** 10.18632/oncotarget.4201

**Published:** 2015-06-17

**Authors:** Laila Siam, Annalen Bleckmann, Han-Ning Chaung, Alexander Mohr, Florian Klemm, Alonso Barrantes-Freer, Raquel Blazquez, Hendrik A. Wolff, Florian Lüke, Veit Rohde, Christine Stadelmann, Tobias Pukrop

**Affiliations:** ^1^ Department of Neurosurgery, University Medical Center, Göttingen, Germany; ^2^ Department of Hematology and Medical Oncology, University Medical Center, Göttingen, Germany; ^3^ Department of Medical Statistics, University Medical Center, Göttingen, Germany; ^4^ Department of Neuroradiology, University Medical Center, Göttingen, Germany; ^5^ Institute of Neuropathology, University Medical Center, Göttingen, Germany; ^6^ Department of Radiotherapy and Radiation Oncology, University Medical Center, Göttingen, Germany; ^7^ Department of Internal Medicine III, Hematology and Medical Oncology, University Hospital Regensburg, Regensburg, Germany

**Keywords:** astrocytes, brain metastasis, glial-pseudo capsule, metastatic infiltration, organ-specific defense

## Abstract

The current approach to brain metastases resection is macroscopic removal of metastasis until reaching the glial pseudo-capsule (gross total resection (GTR)). However, autopsy studies demonstrated infiltrating metastatic cells into the parenchyma at the metastasis/brain parenchyma (M/BP)-interface. Aims/Methods: To analyze the astrocyte reaction and metastatic infiltration pattern at the M/BP-interface with an organotypic brain slice coculture system. Secondly, to evaluate the significance of infiltrating metastatic tumor cells in a prospective biopsy study. Therefore, after GTR, biopsies were obtained from the brain parenchyma beyond the glial pseudo-capsule and analyzed histomorphologically. Results: The coculture revealed three types of cancer cell infiltration. Interestingly, the astrocyte reaction was significantly different in the coculture with a benign, neuroectodermal-derived cell line. In the prospective biopsy study 58/167 (34.7%) samples revealed infiltrating metastatic cells. Altogether, 25/39 patients (64.1%) had proven to exhibit infiltration in at least one biopsy specimen with significant impact on survival (OS) (3.4 HR; *p* = 0.009; 2-year OS was 6.6% versus 43.5%). Exceptionally, in the non-infiltrating cohort three patients were long-term survivors. Conclusions: Metastatic infiltration has a significant impact on prognosis. Secondly, the astrocyte reaction at the M/BP-interface is heterogeneous and supports our previous concept of the organ-specific defense against metastatic (organ-foreign) cells.

## INTRODUCTION

Current neurosurgical and neuropathological guidelines are based on the assumption that cerebral metastases exhibit well-defined margins delimited by a glial pseudo-capsule and are thus locally non-infiltrative in contrast to malignant glioma. In the neurosurgical practice this notion results in a so-called gross total resection (GTR) approach, which consists of the macroscopic removal of the metastatic tissue until reaching the glial pseudo-capsule. Also the neuropathological diagnosis does not include a systematic report on the adjacent brain parenchyma, supposedly reactive benign tissue, despite its potential impact on clinical procedures and prognosis, as exemplified in liver metastasis of colon cancer the resection status has impact on both. On the one hand a prospective biopsy study of brain metastasis supports the current standard approach where in twelve patients non-infiltration beyond the resection margins after GTR was observed [1]. On the other hand retrospective studies in human autopsies [2–4] as well as our own retrospective immunohistochemistry (IHC) studies [5, 6] have challenged this assumption by revealing the presence of infiltrating metastatic tumor cells beyond the glial pseudo-capsule. Furthermore, a retrospective surgical study demonstrated lower local recurrence rates and time to tumor progression after microscopic total resection (MTR), which includes, in addition to GTR, an intraoperative confirmation of metastasis free resection margins by fresh frozen tissue samples [7].

Despite this growing body of evidence demonstrating the presence of metastatic infiltration beyond the glial pseudo-capsule and its potential clinical implications, the result of the above mentioned prospective biopsy study might explain that the GTR-based neurosurgical approach and the neuropathological diagnosis of brain metastasis without standardized reports of the adjacent brain tissue is still the clinical routine. Additionally, this current strategy has impeded a systematic analysis of the human M/BP-interface. In this context, studies from our group and others have indicated that the colonization of the CNS by metastatic cells could be promoted by the cells of the organ-specific defense system of the adjacent brain parenchyma. In short, astrocytes and microglia accumulate at the M/BP-interface, form multiple protrusions and interactions with the immortalized benign non-CNS epithelial cells MDCK and subsequently induce apoptosis in these cells in an *ex-vivo* organotypic brain slice coculture model [8]. However, malignant carcinoma cells do not only appear to withstand this glial-defense at the M/BP-interface, but also misused it to infiltrate the adjacent brain parenchyma [5]. Most importantly, the infiltration of carcinoma cells was prevented by depletion of microglia *in vitro* [6, 9]. Although the mechanisms behind this glial-assisted malignant invasion at the M/BP-interface remain unknown, the mechanisms to counteract this glial-defense seem to be a prerequisite for colonization of the brain parenchyma *in vivo* [10]. Furthermore, an impact of astrocytes on tumor cell proliferation and chemotherapy resistance has also been reported [11–13].

Taken together, these findings suggest that the ability to overcome the glial-defense at the M/BP-interface and infiltrate the adjacent brain parenchyma beyond the glial pseudo-capsule could represent a biological marker with regard to the aggressiveness of the metastatic disease. Thus, in this present study we compared different epithelial cell types (benign and malignant) and the corresponding glial-defense at the M/BP-interface using the above described *ex vivo* model of metastatic infiltration into brain parenchyma. In parallel, we performed a prospective surgical study in patients with resectable brain metastases to determine the degree of infiltration beyond the glial pseudo-capsule for different primaries, using biopsies of the resection margin after GTR.

## RESULTS

### Different infiltration types and glial-reaction are observed in the *in vitro* organotypic brain slice coculture system

**Figure 1:**
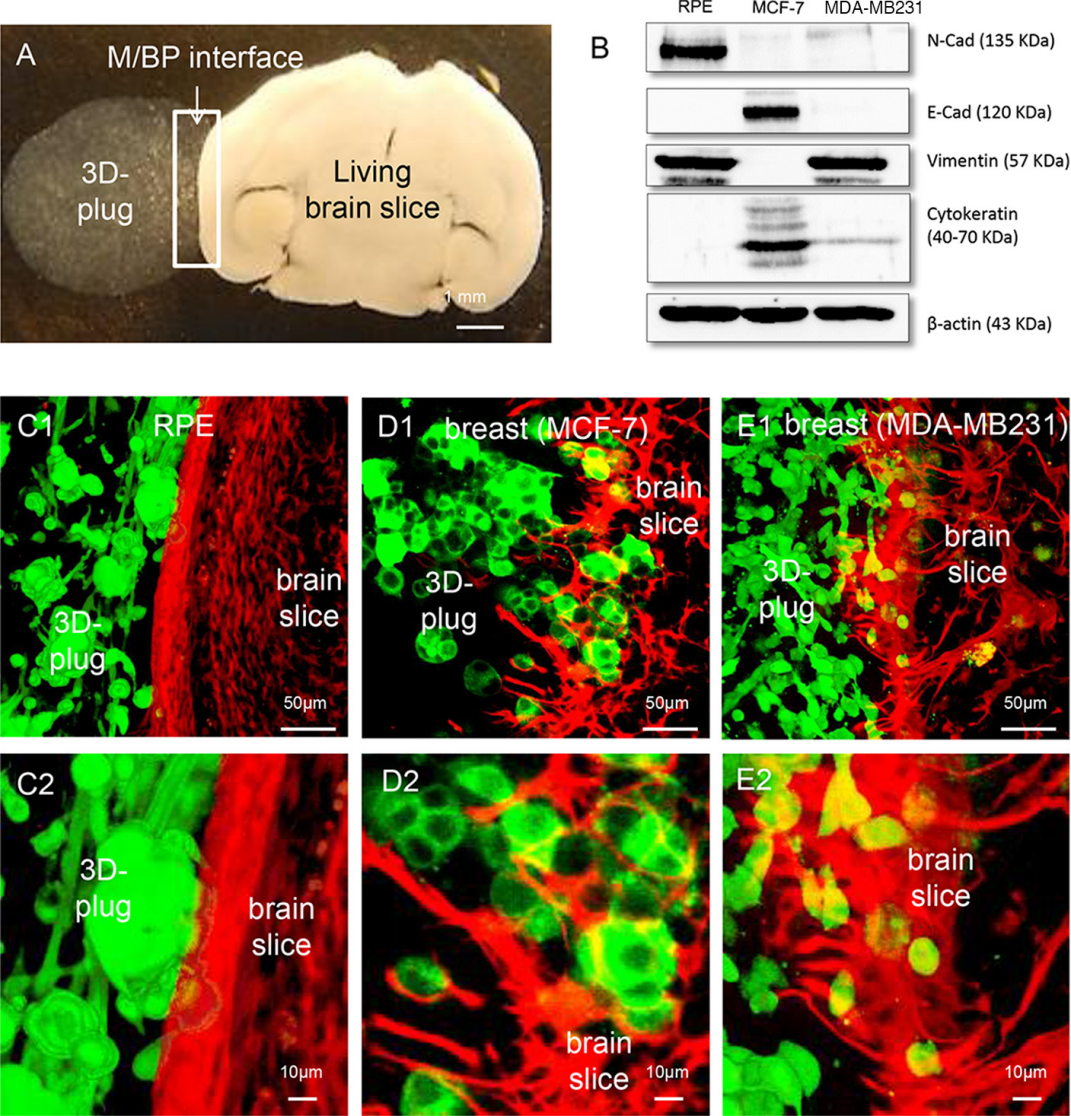
Coculture system with different cancer cells **A.** Macroscopic picture of the coculture system: An organotypic living whole brain slice is cocultured with a 3D tumor plug of MDA-MB231 cells embedded in matrigel (white arrows mark the area of M/BP-interface). **B.** Characterization of the epithelial cells and their mesenchymal characteristics by N-Cadherin (N-Cad), E-Cadherin (E-Cad), Pan-Cytokeratin and Vimentin expression, respectively (see also supplementary Figure 1). **C.** The RPE cell line demonstrates no infiltration (type 0). Interestingly, the RPE also does not cause astrogliosis and astrocyte activation within the otherwise observed defense-reaction. **D.** As already shown, the luminal A breast cancer cell line MCF-7 infiltrates with tumor strands and cohorts (type I) with a significant astrocytic activation. **E.** In contrast, the basal-like breast cancer cell line infiltrates with single cells and mini-spheres (type II) also with a significant astrocytic activation.

Recently, we established an *ex-vivo* organotypic brain slice coculture model to investigate the infiltration of carcinoma cells into adjacent brain parenchyma and the reaction of the glial-defense system against the infiltrating carcinoma cells at the M/BP-interface (Figure 1A) [8]. In these previous studies we used two human breast cancer cell lines, MCF-7, which represents an estrogen (ER) positive cell line, with almost no metastatic potential in animal models. As a second model, we used the SKBR-3 cell line, a her2-neu overexpressing breast cancer cell line, with moderate capacity to metastasize *in vivo*. However, both infiltrated with clusters and cohorts into the adjacent brain parenchyma with support of the glial cells [6, 9]. In order to better understand this glial-facilitated invasion we additionally studied the non-malignant cell line MDCK. This cell line derived from canine kidney epithelial cells of mesodermal origin. The results demonstrated, in contrast to the breast cancer cells (MCF-7, SKBR-3 or 410.4), no evidence of infiltration, however showing a comparable glial-activation. Moreover, the glia-MDCK interactions at the M/BP-interface induced apoptosis of the MDCK cells, while MDCK cells far away from the M/BP-interface without glial contact survived [5]. Thus, we concluded that the actual glial reaction is a generic mechanism and serves to fight various CNS intruders as a defense system. However, this well-intended defense might be misused by carcinoma cells to infiltrate the brain parenchyma [5, 6, 9].

In a first step in this current study we wanted to reveal potential other types of infiltration as well as glial-activation. One the one hand we investigated the MDA-MB231, a basal-like breast cancer cell line, with aggressive metastatic behavior *in vivo*. On the other hand, we chose the RPE cell line, first because the RPE is a human benign, non-metastatic immortalized retinal epithelial cell line. Secondly, it is one of the few epithelial cell lines which are of neuroectodermal origin. Moreover, the retina pigment epithelium has a comparable barrier function like the blood brain barrier and in case of infectious diseases regulates the recruitment of peripheral immune cells. Therefore the RPE cells are also part of the defense-line of the CNS. Considering our previous hypothesis that the glial-reaction is a general orchestrated defense mechanism against CNS-foreign intruders (e.g. epithelial cells from other organs or microbes which are flooded into the CNS by the blood stream during an organ-injury or -infection elsewhere) we speculated that the contact with the RPE cells of neuro-ectodermal origin would not activate the glial defense system at the M/BP-interface in the same way as the CNS-foreign epithelial cells MDCK, MCF-7 or SKBR-3. As control for our experiments we used the MCF-7.

As expected, despite its mesenchymal morphology and molecular parameters (Supplementary Figure 1, Figure 1B) the non-malignant CNS epithelial cell line RPE was unable to infiltrate the brain parenchyma (type 0) (Figure 1C). However, more important the glial-defense behaved like brain slices without any coculture with no obvious signs of glial response. This is a significant difference to the previously described coculture results with the CNS-foreign epithelial cell lines, the benign MDCK, and the breast cancer cell lines MCF-7 and SKBR-3 [5, 6, 9]. Our current results of the MCF-7 confirmed previous results and demonstrated on the one hand a cluster/cohort infiltration (type I) [17, 18], and on the other hand massive glial activation (Figure 1D). Finally, the most aggressive and also *in vivo* very metastatic breast cancer cell line, MDA-MB231, with mesenchymal characteristics and basal-like phenotype (Supplementary Figure 1 and Figure 1B), revealed another type of infiltration in the coculture system. In contrast to the MCF-7 and SKBR-3, the MDA-MB231 infiltrated as single cells or mini-spheres (type II) (Figure 1E). Taken all results together, we identified at least three types of infiltration: type 0 (non-infiltrating), type I (cluster/cohort infiltration), and type III (single cell/mini-sphere infiltration). Moreover, the results of the RPE cell line support our concept of the organ-specific defense against CNS-foreign (metastatic tumor) cells.

### Prospective surgical biopsy study: Evidence of non-infiltrating and infiltrating metastatic tissue

In a next step and as a consequence of the above observations of different invasion types: non-infiltrating benign MDCK and RPE, cluster/cohort infiltration of the low/moderately aggressive MCF-7 and SKBR-3, and the infiltration with single cells/mini-spheres of the very aggressive basal-like MDA-MB231; we assumed a correlation between infiltration and aggressiveness. To investigate this hypothesis and its clinical relevance, we performed a prospective surgical study where after GTR of brain metastasis bioptic tissues from brain parenchyma beyond the pseudo capsule were taken and histomorphologically analyzed. Thirty-nine patients with resectable brain metastases in non-eloquent brain areas were included in this prospective study. A total of 167 biopsies were obtained from the resection cavity wall with a median of three biopsies per patient. The morphological characteristics of the metastatic tumor were determined by routine histological and IHC examination. A total of 20 non-small cell lung cancer (NSCLC), 4 small cell lung cancer (SCLC), 4 carcinomas of the breast, and 4 renal as well as 1 colorectal cancer metastases were included. Additionally, 6 patients with cerebral metastasis of malignant melanoma were studied.

**Figure 2:**
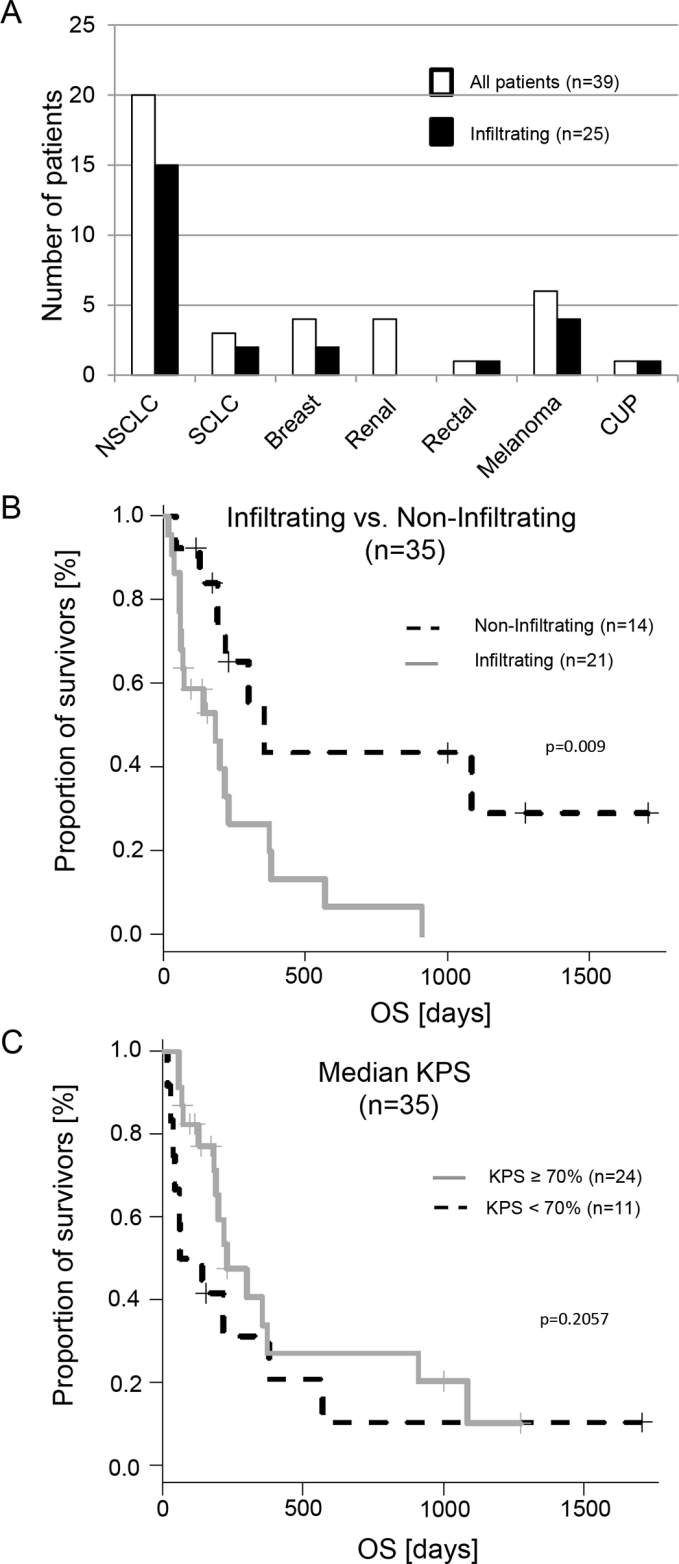
Histological distribution of the investigated samples and impact of the metastatic infiltration on overall survival (OS) **A.** White bars represent the total patient numbers, black bars are the patients with infiltration detected in the cavity wall after GTR. **B.** The gray line represents patients with infiltrated metastatic cells in at least one biopsy specimen, the dashed line those without infiltration. **C.** This diagram shows patients with a KPS ≥ 70% and < 70%. In contrast to the infiltration there is no significant difference.

To determine the presence of infiltrating tumor cells in the adjacent brain parenchyma the brain biopsy specimens were immunohistochemically assessed with appropriate labelling for pan-cytokeratin (NSCLC, breast-, kidney and colorectal carcinoma), Melan-A (melanoma) or chromogranin A (SCLC), based on the immune reactivity of the tumor bulk. According to the presence or absence of metastatic tumor cells beyond the glial pseudo-capsule, the cohort of 39 patients was divided in two groups referred to as a) infiltrating (*n* = 25 patients) and b) non-infiltrating cohort (*n* = 14 patients), respectively. In the infiltrating group, 25 individuals (64.1%) exhibited metastatic tumor infiltration in at least one sample. While the remaining 14 patients presented with a merely displacing non-infiltrating growth pattern in a total of 66 biopsies (Figure 2A) (with a median of 3.5 biopsies/patient in the non-infiltrating group). Overall, the presence of metastatic cells in the adjacent brain parenchyma was observed in 58 out of 167 (34.7%) biopsy specimens obtained from the resection cavity walls.

### Infiltration of metastatic tumor cells correlates with an unfavorable prognosis

In order to investigate our hypothesis of the correlation between infiltration and aggressiveness of the metastatic cells, we calculated the prognostic power of the infiltrating cohort and the non-infiltrating cohort. Prior to this analysis, we excluded four patients of the n = 25 patients in the cohort with infiltrating metastatic tumor cells: one breast cancer patient with so far undetected leptomeningeal spread at the time of surgery and subsequent short OS (47 days) and three NSCLC patients; one due to treatment related mortality two days after surgery and two with a KPS < 50%. Thus 21 patients remained in the infiltrating cohort. In the non-infiltrating cohort all 14 patients fulfilled inclusion criteria. The median follow up of the 35 patients was 171 days [interquartile range (ICR) 69.0 – 327.0]. Patients with evidence of infiltrating growth of metastatic tumor cells showed a significantly shorter OS (*p* = 0.009) (Figure 2B and Table 1). This was even true when the melanoma cases, known for their poor overall survival, were excluded (*n* = 29; *p* = 0. 015).

**Table 1:** Univariate analysis of clinicopathological baseline data affecting survival

Parameter	Classification	Distribution	Impact on survival Hazard Ratio [95%-CI]	Impact on survival *P*-value (logrank)
**Age**	≥ 63.2	51.4% (18/35)	≥ 63.2: 1.89 95% CI [0.84–4.17]	0.1105
	< 63.2	48.6% (17/35)		
**Gender**	female (%)	31.4% (11/35)	male: 2.3 95% CI [0.93–5.71]	0.06761
	male (%)	68.6% (24/35)		
**Number of cerebral metastases**	=1 (%)	54.3% (19/35)	> 1: 1.01 95% CI [0.46–2.23]	0.9826
	> 1 (%)	45.7% (16/35)		
**Secondary organ metastasis at time of diagnosis**	yes (%)	48.6% (17/35)	no: 1.36 95% CI [0.60–3.08]	0.4591
	no (%)	51.4% (18/35)		
**Radiotherapy of the brain (RT) after diagnosis of brain metastasis**	yes (%)	80.0% (28/35)	yes: 1.03 95% CI [0.35–3.02]	0.9603
	no (%)	20.0% (7/35)		
**Chemotherapy (CT) after diagnosis of brain metastasis**	yes (%)	51.4% (18/35)	yes: 1.10 95% CI [0.50–2.44]	0.8109
	no (%)	48.6% (17/35)		
**Proliferation index Ki67**	high ≥ 10 (%)	14.7% (5/34*)	low: 1.55 95% CI [0.57–4.21]	0.3866
	low < 10 (%)	85.3% (29/34*)		
**KPS**	high ≥ 70 (%)	65.7% (23/35)	low: 1.67 95% CI [0.75–3.75]	0.2057
	low < 70 (%)	34.3% (12/35)		
**Infiltration**	yes (%)	62.9% (22/35)	yes: 3.3 95% CI [1.27–8.38]	**0.009717**
	no (%)	37.1% (13/35)		

Moreover, the calculated time to 50% at risk for patients with infiltrating metastatic growth was 117 days 95%-CI [23.3-732.0], and for patients with non-infiltrating growth 230 days 95%-CI [64.0-1575.7] (Wilcox *t*-Test, *p* = 0.01847). Of note, the Hazard ratio for patients with infiltration was 3.4 95%-CI [1.27-8.38], and the 2-year OS was 6.61% 95%-CI [1.01%-43.24%]. In contrast, patients with a non-infiltrating growth pattern had a 2-year OS of 43.5% 95%-CI [21.4%-88.3%]. All long-term survivors belonged to the non-infiltrating group despite suffering from metastatic diseases of different types of primary tumors. For instance, one patient suffering from renal carcinoma had a last follow-up at 2.7 years after surgery, one breast cancer patient at 3.5 years after surgery and one NSCLC patient at 4.7 years after surgery.

Apart from the KPS (*p* = 0.034) (Table 1) none of the other analyzed clinical and diagnostic parameters differed between the infiltrating and non-infiltrating groups (Supplementary Table 1), and none of them had any prognostic value. For this purpose we investigated age, gender, number of cerebral metastases or metastasis at other sites as well as the Ki67 proliferation index and adjuvant whole brain radiation (WBRT) or chemotherapy. Additionally, even the routinely performed MRI scans and analyzed parameters did not reveal any correlation between the two cohorts (Supplementary Table 1). However, the MRI parameters were analyzed retrospectively and future prospective MRI or PET studies are warranted to predict the infiltration status of the metastatic disease.

However, these data again emphasize the undisputed value of the KPS which had a negative correlation −0.376 [95-CI −0.630 to −0.0492] with infiltration (*p* = 0.026). Additionally, calculating the Cox Proportional Hazards Regression resulted in a significant result at *p* = 0.009 for the KPS. However, if we divided the KPS according to the median into low KPS < 70% and high KPS ≥ 70% we got a non-significant HR of 1.67 [95% CI 0.75–3.75] for low KPS < 70% (*p* = 0.206) (Figure 2C). Thus, in contrast to the binary infiltration status (non-infiltration versus infiltrating cohort), the KPS (we included patients with 50–100% in our calculations), does not allow for separation of this patient cohort.

### Histological findings: distinct tumor types differ in their ability to infiltrate and morphological properties of invasion

After establishing the role of infiltrating growth as a predictor of significantly reduced OS, we further asked whether we could describe different types of infiltration in brain metastasis of different primaries like in the organotypic coculture.

### NSCLC samples are predominantly infiltrating

**Figure 3:**
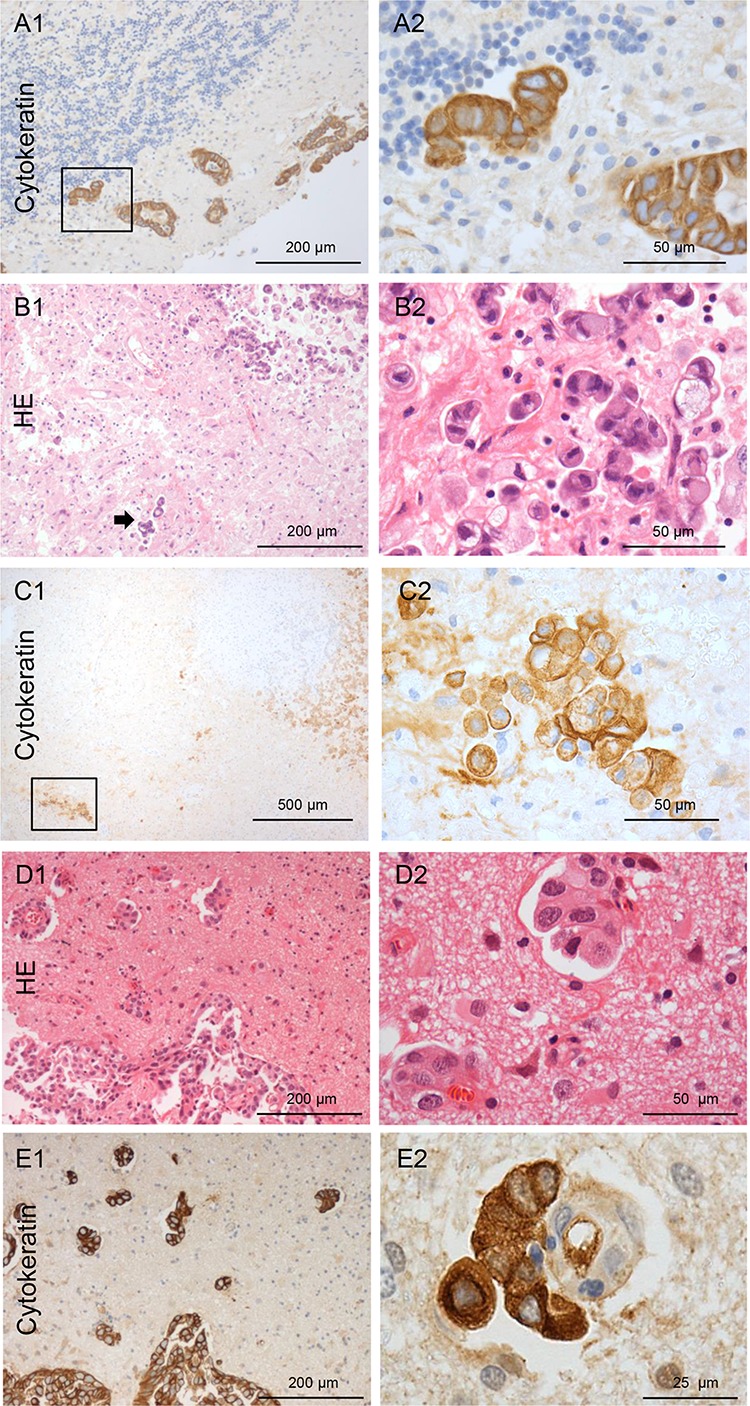
Types of NSCLC carcinoma infiltration **A.** Type I cohort infiltration: Pan-cytokeratin IHC of the infiltrating NSCLC carcinoma cells in the cerebellum with glandular formation (100x and 400x magnification). **B.** Type II single cell and mini-sphere infiltration: HE staining of the infiltrating NSCLC carcinoma cells in the cortex, the arrow marks deeply infiltrated carcinoma cells. **C.** Micrometastasis of a completely resected NSCLC in almost 2 mm distance of the resection cavity measured from the infiltrating edge of the biopsy (on the right: higher magnification of the rectangle). Infiltration type of breast cancer: **D.** Infiltrating tumor strands combined with type I cohort infiltration: HE staining of the breast cancer biopsy tissue (100x and 400x magnification). **E.** Pan-cytokeratin IHC of the infiltrating breast cancer carcinoma cells. The higher magnification demonstrates a cluster of breast cancer cells closely aligned to the external surface of a blood vessel in the Virchow space far beyond the resection margin and pseudo-capsule. (100x and 400x magnification).

The majority of our patient cohort with resection of brain metastasis patients were diagnosed with NSCLC (*n* = 20). Of these 15 (75%) patients had infiltrating carcinoma cells in the adjacent brain parenchyma after GTR. The five patients with non-infiltrating metastatic growth (type 0) demonstrated well-defined metastases margins and a very sharp border at the M/BP-interface, as already described in autopsy studies [2, 3]. In contrast, the samples with brain parenchyma infiltration revealed at least two different patterns. On the one hand, we observed a collective infiltration of metastatic NSCLC cells in cluster/cohorts like MCF-7 (type I) (Figure 3A); and on the other hand, infiltration as single cells and mini-spheres (type II) (Figure 3B–3C). Again, comparable to MDA-MB231 the samples by single cell/mini-sphere infiltration demonstrated deep infiltration into the benign brain parenchyma (Figure 3C). Taken together, NSCLC samples exhibited a wide range of infiltration varying from non-infiltrating up to deep infiltration of > 2 mm, and exhibited at least three infiltration types 0, I and II as observed in the coculture results above.

### Three types of infiltration in breast cancer patients

In two of the four breast cancer patients (50%) who had brain metastasis resected, revealed an infiltrating growth into the brain parenchyma (Figure 3D–3E). One showed a type I cluster/cohort infiltration pattern, where few cohorts of tumor cells were also found to be attached to the external surface of a blood vessels in the Virchow-Robin space far beyond the resection margin and pseudo-capsule (Figure 3E). This is very similar to the attached cohort of MCF-7 cells to a blood vessel in the Virchow-Robin space as shown in the organotypic coculture (Supplementary Figure 2). The second patient revealed type II infiltration with single cells/mini-spheres. This patient was later excluded from the statistical evaluations because of the leptomeningeal spread and subsequent very short OS (47 days) as already described above. The single patient with rectal cancer showed type I (cluster/cohort) infiltration.

### All renal cell cancer (RCC) patients have non-infiltrating growth

**Figure 4:**
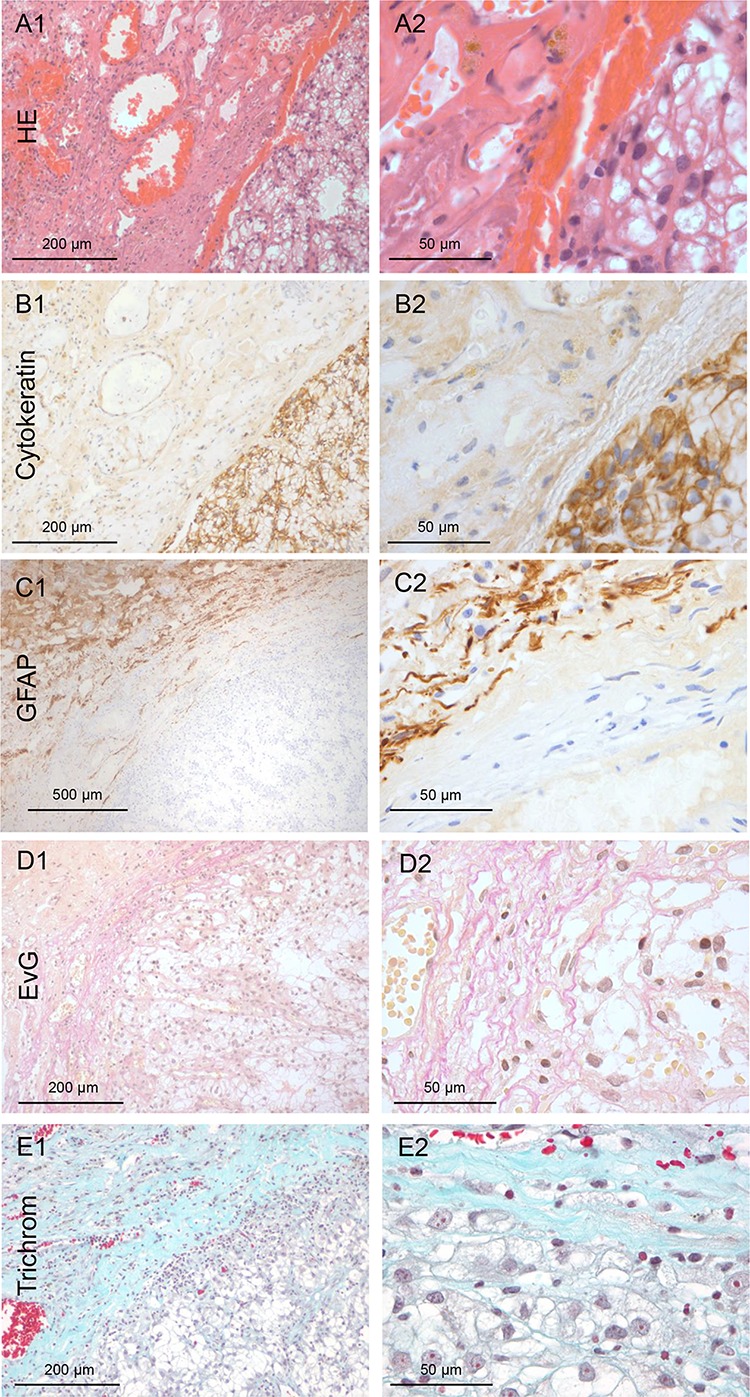
Renal cancer: Type 0 non-infiltration **A.** HE staining of the harvested brain metastasis tissue of renal cancer (100x and 400x magnification). **B.** Pan-cytokeratin IHC of the non-infiltrating renal metastatic cancer mass with a smooth interface to the capsule (100x and 400x magnification). **C.** The GFAP IHC reveals that astrogliosis is not directly adjacent to the metastasis tissue, as is usually the case. **D–E.** Unexpectedly, the Elastica van Gieson (EvG) and the Masson Goldner trichrome staining demonstrated collagen fibers directly adjacent to the tumor cells (red/blue). This indicates that the renal cell cancer forms its own fibrous capsule in the distant organs. (100x and 400x magnification)

In contrast to all other investigated primaries in this patient cohort, non-infiltrating metastatic cells were detected in the corresponding 20 biopsies of the cavity walls of the RCC samples. Microscopy revealed sharply defined margins of the metastatic tissue (Figure 4A–4B). Furthermore, these RCC metastases showed an unique extensive desmoplastic reaction with a fibrous capsule (>1 mm) surrounding the RCC metastasis. In contrast to the typical glial pseudo-capsule, it contained collagen fibers especially at the interface to the metastatic tumor mass (Figure 4D–4E). Thus, the capsule seemed to be composed of at least two layers, the internal collagen-rich layer and the external astrocytic layer (GFAP-staining) (Figure 4C). Moreover, this capsule revealed a high blood vessel density.

### SCLC patients have predominantly type II infiltration

**Figure 5:**
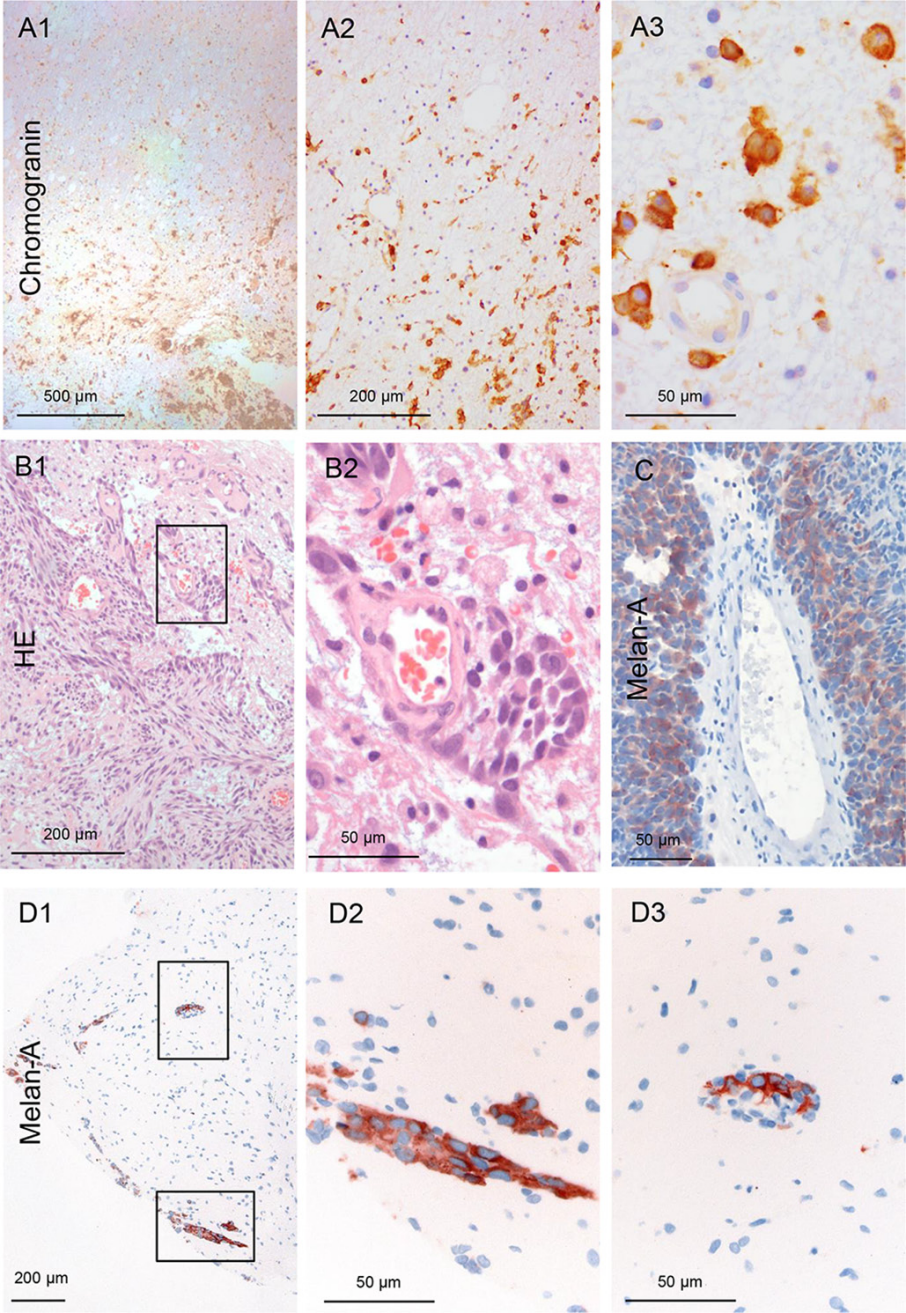
Infiltration of a neuroendocrine CUP **A.** The carcinoma cells demonstrate a type II infiltration (single cell and mini-sphere) (Chromogranin IHC). The entire biopsy tissue is infiltrated with carcinoma cells: thus, the infiltration area is at least 2 mm (100x and 400x magnification). Infiltration of melanoma: **B–D.** The majority of the infiltrating melanoma cells tightly surround blood vessels in these three different patients as sheets/layers. Analogous to the previous *in vivo* description, we named this type III angio-cooptive infiltration. (40x, 100x and 400x magnification).

Four patients were diagnosed with SCLC or neuroendocrine CUP. Only one SCLC patient revealed no evidence of infiltration in four biopsies. The others exhibited a single cell with mini-sphere infiltration (type II) (Figure 5). Most importantly, the infiltration depth exceeded more than 2 mm in some biopsies.

### Metastatic melanoma cells show angio-cooptive infiltration

Six patients had metastases of malignant melanoma, the only non-epithelial cancer in this study. Two patients revealed non-infiltrating metastatic melanoma cells in the biopsies from the resection cavity walls. However, in four cases (66.7%) infiltrating melanoma cells were detected within the biopsies. In contrast to the above described infiltration pattern the majority of the metastatic melanoma cells were seen as sheaths around blood vessels (Figure 5B–5C) protruding far into the adjacent brain parenchyma (Figure 5D1–3). However, this invasion pattern does not correspond to the above described breast cancer patient where only some cohorts were attached to the external surface of the blood vessel in the Virchow-Robin space (Supplemental Figure 2). This type of melanoma infiltration into the brain parenchyma is comparable to the angiotropic invasive subtype of melanoma in primary tumors [14]. Interestingly, these melanoma subtypes are known to develop more frequently brain metastasis and also in a shorter timespan from the onset of the disease than other melanoma subtypes [15]. Until now, we were not able to identify a cell line with a comparable infiltration pattern in our organotypic brain slice model. However, previous live imaging studies of melanoma cell colonization of the brain tissue also demonstrated this angiotropism with growth along the external surface of blood vessels as sheaths in the Virchow-Robin space after extravasation. Concordant with this description [16], we called this subtype III, angio-cooptive infiltration.

## DISCUSSION

**Figure 6:**
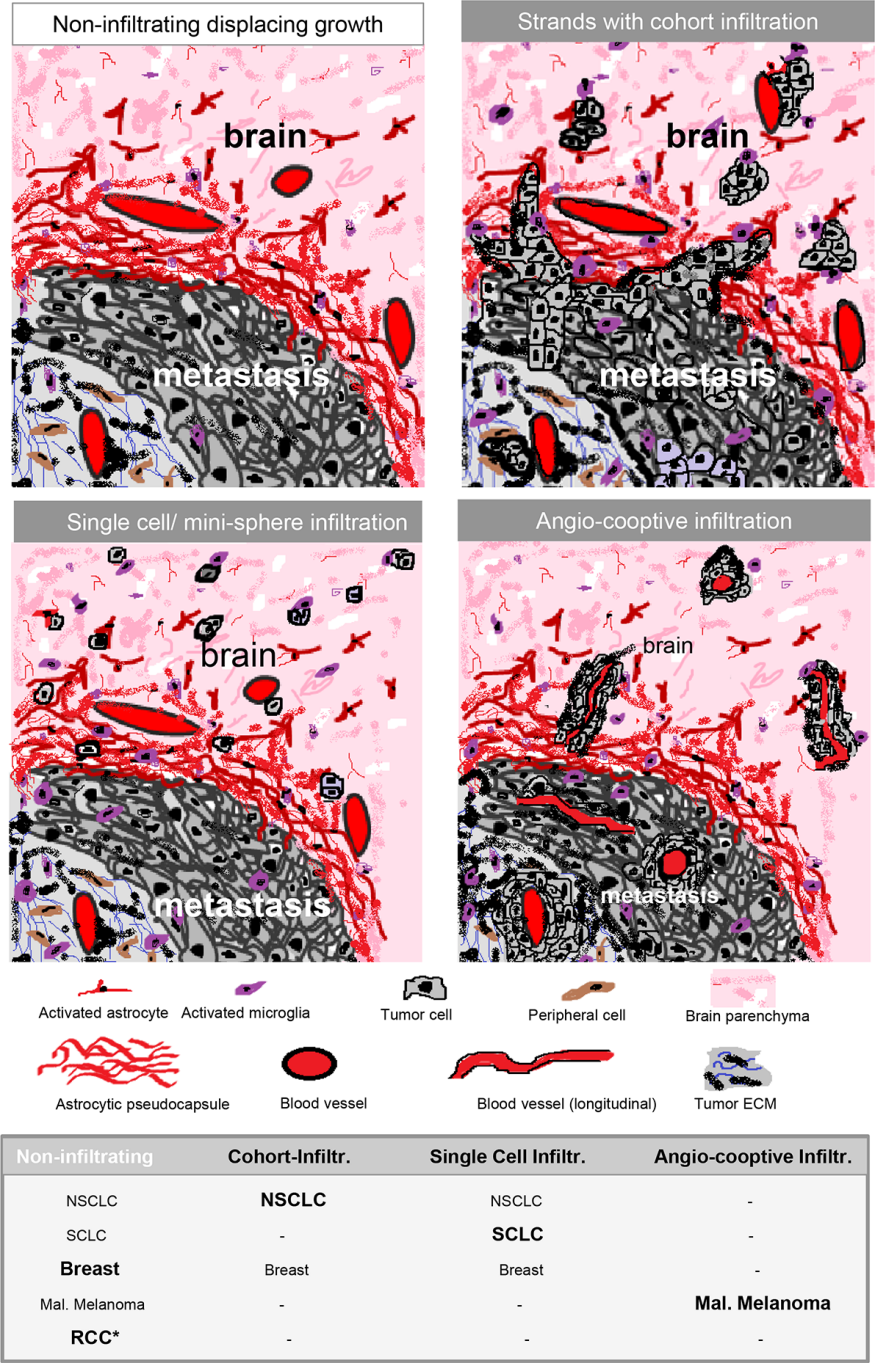
Schemas of different types of infiltration Type 0 = displacing growth without infiltration. Non-infiltrating cancer cells with a significant glial-reaction. Type 1 = cluster-/cohort infiltration: Strands invade into the adjacent brain parenchyma with detached infiltrating cohorts and cluster sometimes found in the Virchow-Robin space, but also without contact to blood vessels. Type 2 = diffuse infiltration: Single cells or mini-spheres (few cells formed together a sphere) infiltrating the brain parenchyma. Type 3 = angio-cooptive: Typically, the infiltration into the adjacent brain parenchyma takes place along pre-existing blood vessels. The table summarizes our results of the biopsy study of NSCLC, SCLC, breast cancer, malignant melanoma and renal cell cancer (RCC) (* with extensive collagen capsule) and visualize the differences in the infiltration. The predominant infiltration pattern is highlighted in bold.

This coculture and prospective biopsy study, not only supports our previously described concept of the organ-specific defense of the glial cells against CNS-foreign metastatic cells during colonization, but also strengthens in our opinion the biological and clinical relevance of the M/BP-interface. Firstly, the metastatic infiltration type at the M/BP-interface has prognostic value. Secondly, there are at least four types of metastatic infiltration at the M/BP-interface (summarized in Figure 6) and thirdly, the primary tumor origin influences the types of infiltration of the metastatic cells. Additionally, the composition and shape of the glial pseudo-capsule is variable and can have tumor-specific characteristics, like the desmoplastic collagen capsule in the RCC samples. However, despite these specific differences in metastatic infiltration and glial pseudo-capsule the M/BP-interface gets almost no attention so far. However, there is increasing evidence that the characteristics of the M/BP-interface obviously could alter the surgical approach, the systematic of the pathology report, the radiosurgery, and finally the choice of the (systemic) treatment modalities.

Recent studies already indicated that the metastatic infiltration pattern or the immune cell infiltration at the tumor margin could have predictive value for biological treatments. For example, an *in vivo* live imaging study revealed that a type I infiltrating lung adenocarcinoma cell line with angiogenic growth responded to anti-VEGF-A therapy, while a type III infiltrating (angio-cooptive) melanoma cell line with sheaths around existing blood vessels did not [16]. A second retrospective IHC analysis of tumor samples, obtained during clinical trials with anti-PD-L-1 immune check point inhibition, demonstrated a negative predictive value of the *excluded immune infiltrate*. The *excluded immune infiltrate* is defined by the accumulation of immune cells at the tumor margins without entering the tumor mass [17]. Thus in patients with brain metastasis the M/BP-interface needs to be analyzed to define this negative predictive biomarker before anti-PD-L-1 treatment. Subsequently, the M/BP-interface should be of special interest in the emerging immunotherapeutic area because the astrocytes are already known to induce drug-resistance. Therefore to manipulate the glial-defense at the M/BP-interface could be an innovative treatment strategy to improve not only chemotherapy but also immunological treatment concepts. Indeed, our previous coculture studies revealed that modulation of the glial-defense by a CXCR4- or WNT-inhibitors or bisphosphonates subsequently reduced the glial-facilitated infiltration of breast cancer cells into the brain parenchyma [5, 6, 9].

### Possible impact on the current guidelines and routine practice

With respect to the presence of infiltrating metastatic cells beyond the resection cavity, our results are in conflict with previously published data where no infiltration was observed. However, we believe that differences in biopsy methods used in both studies are most likely the reason for these differing observations. For instance, by using a tumor alligator forceps instead of a Sudan-Nashold needle [1] we could harvest a larger quantity of adjacent brain parenchyma, thus increasing the likelihood of detection of infiltrating metastatic cells.

A previous retrospective study of patients with singular brain metastases already demonstrated that microscopic total resection (MTR) instead of GTR could be clinically beneficial, at least with regard to lower local recurrence rates. In this work the tissue of the cavity wall was resected beyond the glial pseudo-capsule until tumor-free margins were confirmed by intraoperative fresh frozen sectioning [7]. Also, a second study where the surgeons preoperatively used 5-aminolevulinic acid (5-ALA) for potential intra-vital fluorescence labeling of the metastatic cells revealed residual fluorescence of 5-ALA-positive brain metastases in 75% (24/32) of the patients. Retrospectively, infiltrating tumor cells were histologically confirmed in 6 of 18 samples (33%) obtained from the fluorescent tissue of the cavity walls [18].

To further support our findings as well as differences in the infiltration pattern in dependency to the primary tumor, we combined our results with the SCLC patients from Neves et al. and the results of the second autopsy study [2, 3]. These calculations support our findings: In NSCLC patients 36/49 (73.5%), in SCLC patients 35/44 (79.5%), in melanoma patients 9/12 (75.0%), in breast carcinoma patients 7/13 (53.8%), in CUP 6/6 patients (100%), in gastric/colorectal carcinoma patients 2/4 (50%), and in RCC/urothelial carcinoma patients 0/6 (0%) were identified infiltrating metastatic tumor cells into the adjacent brain parenchyma (see Table in Figure 6). One explanation for the significant difference in RCC patients could be the above described special desmoplastic capsule. This specific collagen capsule in RCC patients could serve as a barrier, which has been already described for the fibrotic capsule in liver metastasis of colon carcinoma [19]. The presence of infiltrating metastatic cells or an extensive desmoplastic capsule (> 1mm), which is highly vascularized, could impact other treatment modalities, for example the dose planning (volume) in stereotactic radiosurgery (SRS). However, further systematic analyses of the extent, content and vascularization of the glial pseudo-capsule and its impact on different treatment approaches (e.g. SRS, anti-VEGF treatment, immune check point modulation) are obviously necessary.

Our results show that Ki-67 has no prognostic value in general, and has recently been confirmed in a huge retrospective IHC study of brain metastasis patients. There low Ki-67 was only prognostic in NSCLC and RCC [20]. We also confirmed the prognostic value in a previous study of a Adenocarcinoma patients of the lung [21]. Therefore, Ki-67 should be routinely included in the pathological report of brain metastasis of RCC and NSCLC patients.

In light of these observations, we are convinced that the use of MTR or eventually intra-vital fluorescence labeling should be further studied in clinical trials, particularly in patients with solitary/single metastasis in non-eloquent brain areas. Furthermore, the pathology report should include the infiltration pattern, the depth of infiltration and eventually the extent and kind of pseudo-capsule if adjacent brain parenchyma is available for histological analysis. In NSCLC and RCC patients Ki-67 seems prognostic and in near future the description of the immune infiltrate at the M/BP-interface could be of negative predictive value for immune checkpoint modulation therapies of melanoma, lung, breast and colon cancer patients with brain metastases.

In summary, our studies underline the biological significance of the M/BP-interface and support the concept of the glial defense against CNS-foreign cell types, including epithelial cells from other organs. Furthermore, our findings point to the future importance of a precise histology report, including the determination of infiltration and composition of the M/BP-interface. Moreover, it strengthens the value of the neurosurgical resection and could lead to the adaption of current guidelines to include the existence of metastatic infiltration, in particular in non-eloquent brain areas. Furthermore, our findings underline the necessity to deepen our understanding of the biology of brain metastasis at the M/BP-interface with its very special microenvironment and unique resident glial-defense system.

## PATIENTS, MATERIALS AND METHODS

### Inclusion criteria and study procedure

Ethical approval of this one-center prospective study was given by the local ethics committee of the University Medical Center Göttingen. After informed consent, patients 1) with a limited number of cerebral metastases, 2) without signs of leptomeningeal spread in magnetic resonance imaging (MRI) and 3) who were scheduled for resection of one to three metastases localized in non-eloquent brain regions were included in this study. After GTR of the metastases, biopsies of grossly normal-appearing brain parenchyma adjacent to the resection cavity were performed with tumor alligator forceps. Where possible, biopsies were taken from the anterior, posterior, medial, lateral, superior, and inferior walls of the resection cavity. The tissue samples were immediately fixed in buffered formalin. The clinical documentation was carried out in the clinical cancer registry of the Göttingen Comprehensive Cancer Center.

### Histological techniques and IHC

The metastatic tissue obtained by GTR was used to determine the probable tissue of origin by morphology and routine IHC investigations. The fixed biopsy specimens taken from the walls of the resection cavity beyond the glial pseudo-capsule were paraffin embedded, de-waxed, rehydrated, cut into 2–5 μm sections using a microtome (Leica SM 2000R, Leica, Wetzlar, Germany) and mounted on glass slides following standard protocols. HE, PAS, Gomori, Elastica van Gieson (EvG) and the Masson Goldner trichrome staining were performed according to routine protocols, and IHC was performed as described previously [22]. Briefly, tissue sections were incubated at 4°C overnight with primary antibodies against Melan-A (1:100, mouse monoclonal, A103), chromogranin A (1:50, mouse monoclonal, DAK-A3) or pan-cytokeratin CK AE 1/3 (1:100, mouse monoclonal, AE1/AE3) diluted in 10% fetal calf serum (FCS) in PBS. Antibody detection was achieved using a biotinylated secondary antibody followed by addition of avidin-peroxidase and developed with 3, 3′-diaminobenzidine hydrochloride (DAB; Sigma, St. Louis, Mo, USA). Both primary and secondary antibodies were purchased from Dako (Glostrup, Denmark). For the microscopic evaluation an Olympus BX41 microscope was used.

### Statistics and bioinformatics

All patients were included to calculate the percentage of patients demonstrating infiltration of metastatic tumor cells in the specimens obtained after GTR. Patients without tumor cell infiltration in any of the biopsies were grouped into the “non-infiltrating” group, whereas patients with at least one biopsy specimen with infiltrating tumor cells belonged to the “infiltrating” cohort. For calculation of the overall survival, only patients with a KPS ≥ 50% and without treatment-related mortality were included. Survival analysis was performed from the date of cerebral metastasis resection until last follow-up using the R package *survival*. Events were defined as cancer-related death; all other events were considered censored. Survival data were visualized using Kaplan–Meier plots, and significance was calculated using the Cox proportional hazards model and log-rank test as described previously [21]. Correlations were calculated using the Pearson's correlation test. The statistical analysis was carried out using the Statistical Computing Software R [Free statistical software R, version 2.12.2, http://www.r-project.org]. Significance for comparison between cohorts (non-infiltrating versus infiltrating) was calculated using Fisher's exact test for categorical variables or variables that were discretized (e.g., gender, type of therapy, MRI results of size, necrosis or contrast-enhancement), and the Wilcoxon test for numeric variables (e.g., age, KPS). *P*-values < 0.05 were considered significant.

### Cell lines and immunoblotting

The human breast cancer cell lines MCF-7 and MDA-MB231 were obtained from the DSMZ and were maintained in RPMI-1640 medium (PAA Laboratories Inc., Cölbe, Germany) and the human retinal pigment epithelium cell line (RPE) in DMEM medium (Biochrome, Berlin, Germany) supplemented with 10% fetal calf serum (FCS, Invitrogen, Karlsruhe, Germany). Stable transfections with the mammalian Turbo GFP vector (FP512, Evrogen Inc., Heidelberg, Germany) were performed with the Nanofectin kit (PAA Laboratories Inc., Cölbe, Germany) in accordance with the manufacturer's protocol, and selection was done using geneticin resistance (G418, Roche Inc, Basel, Switzerland). To obtain homogeneous GFP expression, cells were sorted using FACS (BD FACS Aria II, Heidelberg, Germany). To assess protein expression and the distribution of E-Cadherin, N-Cadherin and Vimentin, we performed immunoblotting (IB) and immunofluorescence staining (IF) with primary antibodies specific to E-cadherin (#610182), N-cadherin (#610921, both from BD Translab), Vimentin (#5741, Cell Signaling), and β-actin (#A5441, Sigma Aldrich). The IB [5] and IF [23] were performed as previously described. For IB, whole cell lysates (40 μg) were processed with 10% SDS-polyacrylamide gel (PAGE) and visualized using enhanced chemiluminescence (ECL, GE Healthcare, Pollards Wood, UK) with the Fuji LAS 4000 image system. For IF, the cells were incubated with the primary antibody (1:100) for 30 minutes at room temperature, followed by anti-mouse-TRITC staining (1:150, sc- 3796, Santa Cruz) or anti-rabbit-TRITC (1:150, sc- 3841, Santa Cruz) for 30 minutes and counterstained with DAPI, cover slipped, and analyzed with a confocal microscope (LSM 510, Zeiss, Göttingen, Germany).

### Organotypic brain cocultures

The organotypic brain slice cocultures were performed with the interface technique following the previously described protocol [8]. MCF-7-GFP, MDA-MB231-GFP or RPE-GFP cells were used for the cocultures with the mouse brain slice cocultures. Tumor cell invasion was evaluated using a Zeiss confocal laser scanning microscope (LSM 510, Zeiss, Göttingen, Germany) as previously described [5, 6, 8].

## SUPPLEMENTARY MATERIAL FIGURES AND TABLE


