# Supplementary material for: The metastatic infiltration at the metastasis/brain parenchyma-interface is very heterogeneous and has a significant impact on survival in a prospective study
Source: Oncotarget. 2015 Jun 17;6(30):29254–67. doi: 10.18632/oncotarget.4201 (PMC4745724; doi:10.18632/oncotarget.4201)
Supplement: Supplementary file 1 [file oncotarget-06-29254-s001.pdf]

SUPPLEMENTARY FIGURES AND TABLE

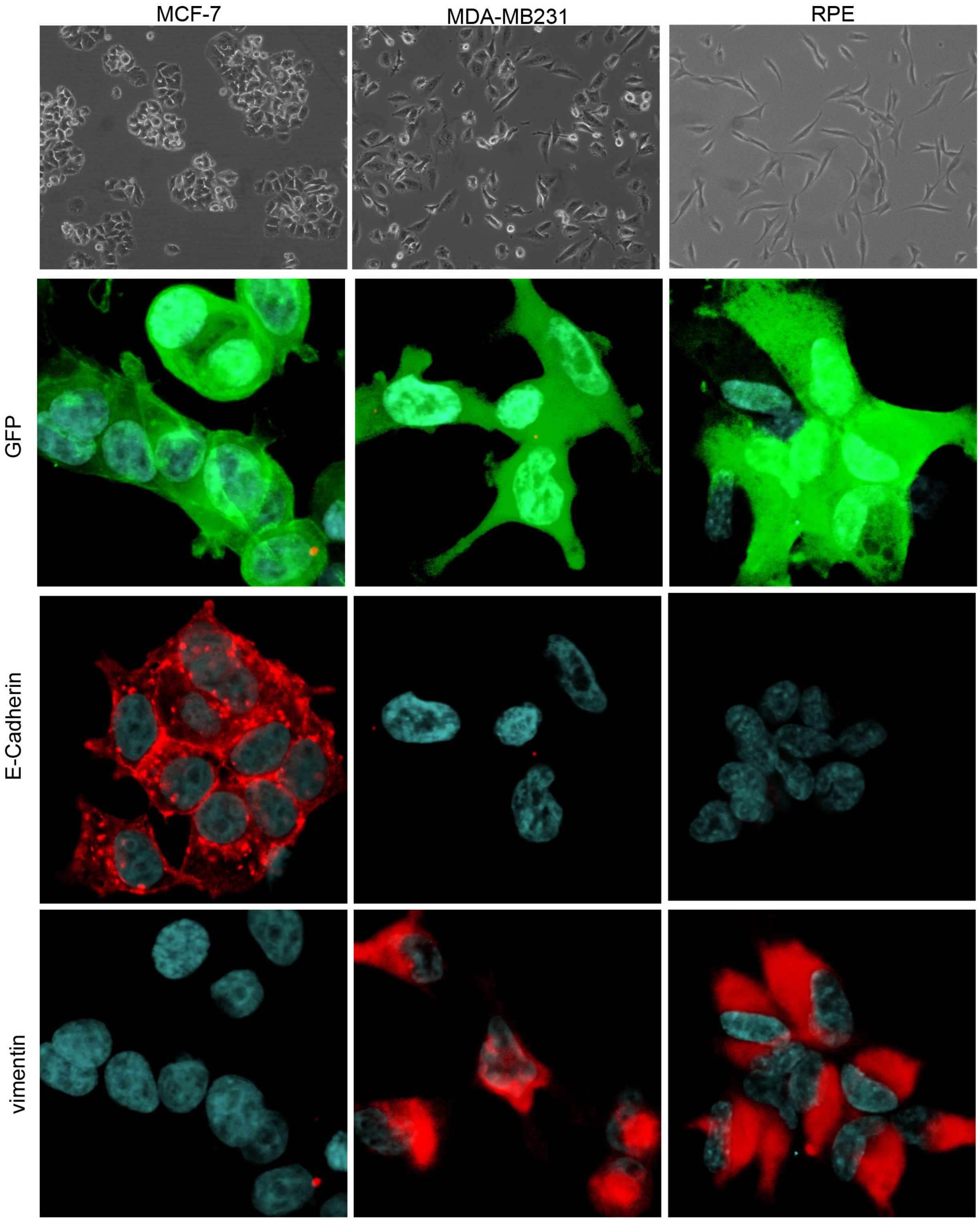

Supplementary Figure 1: Differences in morphology of the epithelial breast cancer cell line MCF-7, the breast cancer cell MDA-MB231 with mesenchymal morphology and epithelial RPE with also mesenchymal morphology and their respective expression of GFP, E-Cadherin, vimentin detected by confocal microscopy.

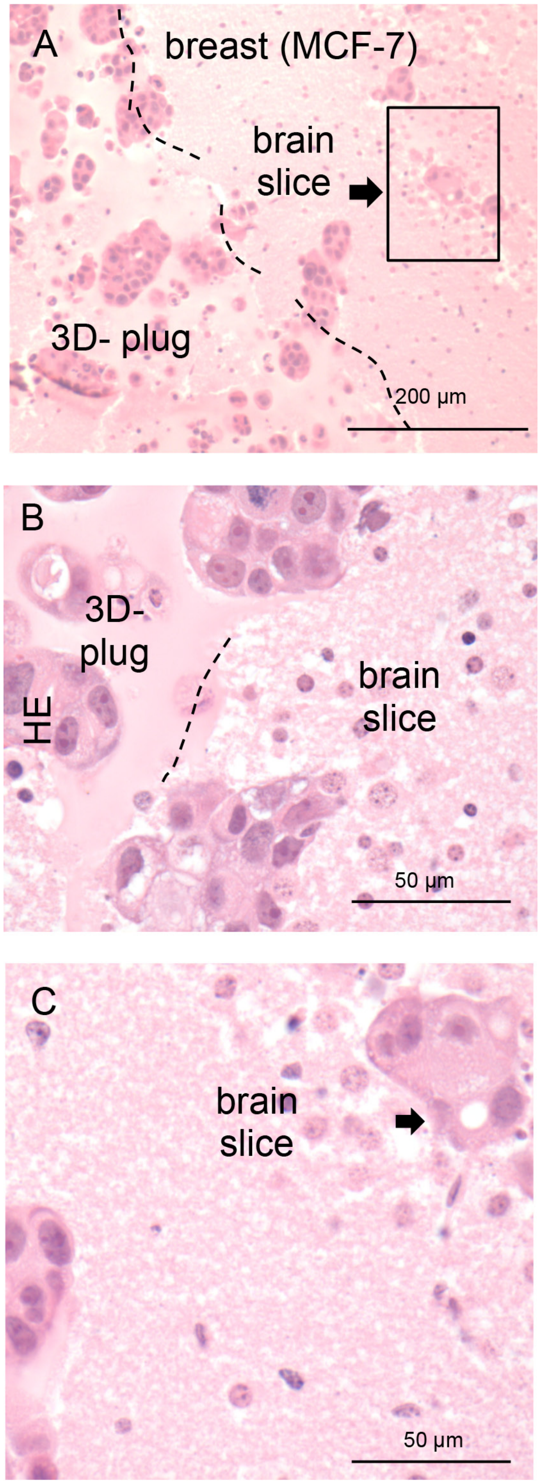

Supplementary Figure 2: A. Overview by HE staining of a co-culture with the breast cancer cell line MCF-7. The interface/boarder is marked by the dashed line. **B.** Typical carcinoma cell strands already invaded the slice and destroyed the slice margins at the interface. **C.** Some cohorts already invaded > 200 µm in the brain slice parenchyma. Remarkably, we also detected breast cancer cells aligned at the external surface of a blood vessel in the Virchow-Robin space (arrow) as in the patient specimens.

Supplementary Table 1: Analyzes of differences of additional analyzed clinical and diagnostic parameters between the infiltrating and non-infiltrating groups

Parameter	Classification	No infiltration	Infiltration	*P*-Value
**Age**	median [95%–CI]	63.6 [95%–CI: 47.5–76.3]	62.8 [95%–CI: 42, 9–80.0]	0.9067
**Gender**	female [%]	14.3 (5/35)	17.1 (6/35)	0.7077
	male [%]	22.9 (8/35)	45.7 (16/35)	
**Number of cerebral metastases**	solitary [%]	20.0 (7/35)	34.3 12/35)	1
	> 1 metastasis [%]	17.1 (6/35)	28.6 (10/35)	
**Secondary organ metastasis at time of diagnosis**	Yes [%]	20.0 (7/35)	28.6 (10/35)	0.7332
	No [%]	17.1 (6/35)	34.3 (12/35)	
**Radiotherapy of the brain (RT) after diagnosis of brain metastasis**	Yes [%]	28.6 (10/35)	51.4 (18/35)	1
	No [%]	8.6 (3/35)	11.4 (4/35)	
**Chemotherapy (CT) after diagnosis of brain metastasis**	Yes [%]	17.1 (6/35)	34.3 (12/35)	0.7332
	No [%]	20.0 (7/35)	28.6 (10/35)	
**Sharp contrast boarders**	Yes [%]	14.1 (5/34*)	29.4 (10/34*)	0.5572
	No [%]	20.6 (7/34*)	35.3 (12/34*)	
**Homogenous contrast enhancement**	Yes [%]	8.8 (3/34*)	8.8 (3/34*)	0.3471
	No [%]	26.5 (9/34*)	55.9 (19/34*)	
**Central necrosis**	Yes [%]	29.4 (10/34*)	55.9 (19/34*)	0.5899
	No [%]	5.8 (2/34*)	8.8 (3/34*)	
**Proliferation index Ki67**	< 10 [%]	8.8 (3/34*)	5.8 (2/34*)	0.3476
	≥ 10 [%]	29.4 (10/34*)	55.9 (19/34*)	
**KPS (Median)**	< 70 [%]	5.7 (2/35)	28.6 (10/35)	**0.03408**
	≥ 70 [%]	31.4 (11/35)	34.3 (12/35)	
